# Azacitidine front-line in 339 patients with myelodysplastic syndromes and acute myeloid leukaemia: comparison of French-American-British and World Health Organization classifications

**DOI:** 10.1186/s13045-016-0263-4

**Published:** 2016-04-16

**Authors:** Lisa Pleyer, Sonja Burgstaller, Reinhard Stauder, Michael Girschikofsky, Heinz Sill, Konstantin Schlick, Josef Thaler, Britta Halter, Sigrid Machherndl-Spandl, Armin Zebisch, Angelika Pichler, Michael Pfeilstöcker, Eva-Maria Autzinger, Alois Lang, Klaus Geissler, Daniela Voskova, Dietmar Geissler, Wolfgang R. Sperr, Sabine Hojas, Inga M. Rogulj, Johannes Andel, Richard Greil

**Affiliations:** 3rd Medical Department with Hematology and Medical Oncology, Hemostaseology, Rheumatology and Infectious Diseases, Laboratory for Immunological and Molecular Cancer Research, Oncologic Center, Paracelsus Medical University Salzburg, Salzburg, Austria; Center for Clinical Cancer and Immunology Trials at Salzburg Cancer Research Institute, Salzburg, Austria; Cancer Cluster Salzburg, Salzburg, Austria; Department of Internal Medicine IV, Klinikum Wels-Grieskirchen, Wels, Austria; Department of Internal Medicine V, Innsbruck Medical University, Innsbruck, Austria; Department of Hematology and Oncology, Elisabethinen Hospital, Linz, Austria; Department of Hematology, Medical University of Graz, Graz, Austria; Department for Hematology and Oncology, LKH Leoben, Leoben, Austria; 3rd Medical Department for Hematology and Oncology, Hanusch Hospital, Vienna, Austria; First Medical Department, Center for Oncology, Hematology and Palliative Care, Wilhelminenspital, Vienna, Austria; Department of Internal Medicine, LKH Feldkirch, Feldkirch, Austria; 5th Medical Department, Hospital Hietzing, Vienna, Austria; Department of Internal Medicine III, General Hospital, Linz, Austria; 1st Medical department, Klinikum Klagenfurt, Klagenfurt, Austria; Department of Internal Medicine I, Division of Hematology and Hemostaseology, Medical University of Vienna, Vienna, Austria; Department of Internal Medicine, LKH Fürstenfeld, Fürstenfeld, Austria; Department of Hematology, Clinical Hospital Merkur, Zagreb, Croatia; Department of Internal Medicine II, LKH Steyr, Steyr, Austria

**Keywords:** AML, MDS, WHO, FAB, Classification, RAEB-t, Bone marrow blast count, Azacitidine, Austrian Azacitidine Registry

## Abstract

**Background:**

The MDS-IWG and NCCN currently endorse both FAB and WHO classifications of MDS and AML, thus allowing patients with 20–30 % bone marrow blasts (AML20–30, formerly MDS-RAEB-t) to be categorised and treated as either MDS or AML. In addition, an artificial distinction between AML20–30 and AML30+ was made by regulatory agencies by initially restricting approval of azacitidine to AML20–30. Thus, uncertainty prevails regarding the diagnosis, prognosis and optimal treatment timing and strategy for patients with AML20–30. Here, we aim to provide clarification for patients treated with azacitidine front-line.

**Methods:**

The Austrian Azacitidine Registry is a multicentre database (ClinicalTrials.gov: NCT01595295). For this analysis, we selected 339 patients treated with azacitidine front-line. According to the WHO classification 53, 96 and 190 patients had MDS-RAEB-I, MDS-RAEB-II and AML (AML20–30: *n* = 79; AML30+: *n* = 111), respectively. According to the FAB classification, 131, 101 and 111 patients had MDS-RAEB, MDS-RAEB-t and AML, respectively.

**Results:**

The median ages of patients with MDS and AML were 72 (range 37–87) and 77 (range 23–93) years, respectively. Overall, 80 % of classifiable patients (≤30 % bone marrow blasts) had intermediate-2 or high-risk IPSS scores. Most other baseline, treatment and response characteristics were similar between patients diagnosed with MDS or AML. WHO-classified patients with AML20–30 had significantly worse OS than patients with MDS-RAEB-II (13.1 vs 18.9 months; *p* = 0.010), but similar OS to patients with AML30+ (10.9 vs 13.1 months; *p* = 0.238). AML patients that showed MDS-related features did not have worse outcomes compared with patients who did not (13.2 vs 8.9 months; *p* = 0.104). FAB-classified patients with MDS-RAEB-t had similar survival to patients with AML30+ (12.8 vs 10.9 months; *p* = 0.376), but significantly worse OS than patients with MDS-RAEB (10.9 vs 24.4 months; *p* < 0.001).

**Conclusions:**

Our data demonstrate the validity of the WHO classification of MDS and AML, and its superiority over the former FAB classification, for patients treated with azacitidine front-line. Neither bone marrow blast count nor presence of MDS-related features had an adverse prognostic impact on survival. Patients with AML20–30 should therefore be regarded as having ‘true AML’ and in our opinion treatment should be initiated without delay.

**Electronic supplementary material:**

The online version of this article (doi:10.1186/s13045-016-0263-4) contains supplementary material, which is available to authorized users.

## Background

Since 1982, patients with 20–30 % bone marrow blasts have been considered to have myelodysplastic syndromes with refractory anaemia and excess blasts in transformation (MDS-RAEB-t) according to the French-American-British (FAB) classification [1]. When the World Health Organization (WHO) classification came into effect in 2001, these patients were considered to have acute myeloid leukaemia (AML) with a low bone marrow blast count (hereafter AML20–30; Additional file 1: Table S1) [1, 2]. This new classification (updated in 2008 [3]) was driven by novel insights from several studies that identified that bone marrow blast count had more prognostic weight than was originally perceived and that MDS-RAEB-t patients had similar outcomes to patients with AML and more than 30 % bone marrow blasts (hereafter AML30+), partly owing to the fact that MDS-RAEB-t commonly transformed into AML [4–12].

Although the sum of available data led the WHO to conclude that AML20–30 (formerly MDS-RAEB-t) and AML30+ were essentially the same disease 15 years ago, several relevant groups do not appear to consider the scientific evidence to be strong enough: (i) The National Comprehensive Cancer Network (NCCN) endorses both FAB and WHO classification systems, allowing MDS-RAEB-t to be diagnosed and treated as either MDS or AML [13, 14]; (ii) many large phase III randomised clinical trials still retain MDS-RAEB-t as an MDS sub-entity [15]; and (iii) while the division of the category MDS-RAEB into RAEB-I and RAEB-II by WHO was validated and generally accepted to add significant prognostic value [16–18], scientific debate regarding the abandonment of the sub-entity MDS-RAEB-t by WHO remains between members of the MDS Study Group [19], the WHO Myeloid Disease Writing and Clinical Advisory Committees [20], and between other renowned experts in the field [21, 22]. Therefore, uncertainty prevails regarding the diagnosis, prognosis, and optimal treatment timing and strategy for patients with AML20–30.

Azacitidine was approved for the treatment of patients with MDS and AML20–30 in 2004 by the Food and Drug Agency (FDA) and in 2008 by the European Medicines Agency (EMA). Although the patient population included in clinical trial protocols resulting in drug approval included up to 38 % of patients with AML30+ (CALGB-protocols 8921 and 9221) [23], both the FDA and the EMA restricted approval of azacitidine to AML20–30, and a further large randomised clinical trial was required to prove the efficacy of azacitidine in AML30+. This artificial distinction between AML20–30 and AML30+ was made by the regulatory agencies and reflects neither the former FAB classification of MDS-RAEB-t (which could also be diagnosed if bone marrow blasts were <20 % with the presence of >4 % peripheral blood blasts or Auer rods) nor the current WHO classification of MDS and AML. Azacitidine treatment for patients with AML30+ was off-label until very recently (30 October 2015), when the EMA extended the indication for azacitidine to include this patient subgroup. Approval was mainly based on the results of a phase III randomised trial performed exclusively in AML30+ patients [24]. While bone marrow blast percentage has retrospectively been analysed in smaller patient cohorts for its potential as a prognostic factor for AML patients treated with azacitidine front-line [25, 26], no in-depth analysis of patient baseline characteristics, treatment characteristics and outcomes exist. To date, no clinical trial has directly compared the efficacy of azacitidine in AML patients with 20–30 % vs >30 % bone marrow blasts, a gap we aimed to bridge.

In this study, we provide the first comparison of baseline characteristics and outcomes of patients with MDS and AML treated with azacitidine front-line, with the intention to (i) provide further insight into the efficacy of azacitidine in the subgroup of AML patients for whom this drug was initially approved (i.e. AML20–30) and to assess whether patients with AML30+ benefit from azacitidine treatment in a similar way to patients with AML20–30; (ii) evaluate the potential prognostic relevance of the presence of MDS-related features (MRF) in AML and (iii) assess the outcomes of patients with MDS and AML as classified by both the WHO and FAB systems, in order to clarify whether elderly, intensive chemotherapy (IC)-ineligible patients with AML20–30 (formerly RAEB-t) should be regarded and treated as having MDS or AML. To answer these questions, this analysis focuses on the differences in morphological features (blast count in peripheral blood and/or bone marrow and presence of dysplasia) between the WHO and FAB classifications.

## Results

### Study cohort

We identified 339 patients from the Austrian Azacitidine Registry (AAR) who had been diagnosed with MDS-RAEB-I/II or AML according to the WHO classification and treated with azacitidine as a front-line agent. Of these, 149 patients had MDS, of which 53 had RAEB-I and 96 had RAEB-II; 190 patients had AML. Among AML patients, 79 had AML20–30 and 111 had AML30+. The median age of patients with MDS was 72 (range 37–87) years, and the median age of patients with AML was 77 (range 23–93) years (Table 1). Only four patients were <40 years, three of which proceeded to allogeneic stem cell transplantation, and one of which died while on treatment with azacitidine (Additional file 2: Table S2). A total of 4, 44, 35 and 4 % of patients with MDS-RAEB-I had a low, intermediate-1, intermediate-2 or high International Prognostic Scoring System (IPSS) risk score, whereas 0, 9, 42 and 34 % of patients with MDS-RAEB-II had a low, intermediate-1, intermediate-2 or high IPSS risk score, and 0, 0, 14 and 85 % of patients with AML20–30 had a low, intermediate-1, intermediate-2 or high IPSS risk score, respectively. This score is not applicable to AML patients with more than 30 % bone marrow blasts. Of patients diagnosed with WHO-AML, 7, 4, 71 and 19 % had therapy-related AML, AML with recurrent cytogenetic abnormalities, AML-MRF and AML not otherwise specified, respectively.

**Table 1:** Comparison of baseline characteristics of patients with WHO-MDS or WHO-AMLreceiving azacitidine front-line

	AML30+ (*n* = 111)	AML20–30 (*n* = 79)	*p* value	AML20–30 (*n* = 79)	RAEB-II (*n* = 96)	*p* value	RAEB-II (*n* = 96)	RAEB-I (*n* = 53)	*p* value
AZA first-line, %	100	100	1	100	100	1	100	100	1
Age (years), median (range) Age ≥75 years, %	77 (23–93) 57.7	77 (44–93) 59.5	1 0.867	77 (44–93) 59.5	72 (37–87) 38.5	0.682 0.034	72 (37–87) 38.5	71 (42–87) 41.5	0.933 0.737
Male, %	55.0	65.6	0.334	65.6	65.6	1	65.6	77.4	0.323
MRF^a^, %	66.7	78.5	0.327	78.5	100^b^	0.110	100^b^	100^b^	1
Therapy-related, %	4.5	8.9	0.229	8.9	9.4	0.906	9.4	11.3	0.677
PB-blasts (%), median (range) ≥0 %, %	4 (0–86) 65.8	2 (0–58) 64.6	0.414 0.916	2 (0–58) 64.6	1 (0–19) 44.8	0.564 0.058	1 (0–19) 44.8	0 (0–4) 37.7	0.317 0.434
ECOG PS, % Grades 0–1 Grade 2 Grades 3–4	67.6 23.4 9.0	69.6 24.2 6.3	0.865 0.909 0.490	69.6 24.2 6.3	80.2 16.7 3.1	0.386 0.241 0.297	80.2 16.7 3.1	83.0 11.3 5.7	0.827 0.308 0.381
IPSS cytogenetic risk group^c^, % Good Normal karyotype Intermediate Poor Not evaluable	50.5 45.1 14.4 21.6 13.5	46.2 38.5 21.8 20.5 11.5	0.602 0.470 0.219 0.865 0.689	46.2 38.5 21.8 20.5 11.5	48.9 45.7 11.7 30.9 7.3	0.781 0.433 0.081 0.147 0.333	48.9 45.7 11.7 30.9 7.3	45.3 30.2 20.8 24.5 9.4	0.710 0.075 0.110 0.390 0.607
RBC-TD, %	55.9	48.1	0.444	48.1	55.2	0.485	55.2	67.9	0.252
PLT-TD, %	27.9	21.5	0.363	21.5	31.3	0.177	31.3	22.6	0.236
Hb (g/dL), median (range)	9.1 (5.8–14.2)	9.1 (6.3–13.4)	1	9.1 (6.3–13.4)	9.1 (6.7–14.2)	1	9.1 (6.7–14.2)	8.9 (2.5–15)	0.964
PLT (G/L), median (range)	50 (7–1270)	66 (6–1100)	0.137	66 (6–1100)	44 (7–1184)	0.036	44 (7–1184)	51 (8–610)	0.473
WBC (G/L), median (range)	2.5 (0.6–96.0)	2.5 (0.6–41.6)	1	2.5 (0.6–41.6)	2.5 (0.8–96.0)	1	2.5 (0.8–96.0)	2.5 (0.6–13.8)	1
ANC (G/L), median (range)	0.5 (0–37.2)	0.7 (0–28.0)	0.856	0.7 (0–28.0)	0.9 (0–42.0)	0.874	0.9 (0–42.0)	1.1 (0.2–10.9)	0.888

^a^MRF: MDS-related features, as defined by presence of MDS-related cytogenetics (MRC) and/or antecedent haematological disease (AHD) and/or myelodysplasia

^b^For the diagnosis of MDS according to WHO, the presence of myelodysplasia is required in all patients (i.e. 100 %)

^c^The IPSS cytogenetic risk score was established for and validated in patients with MDS and is not commonly used to stratify cytogenetic risk in AML patients. However, we used this score for both MDS and AML patients, in order to be able to compare frequencies of karyotypes across these patient groups

*WHO* World Health Organization, *MDS* myelodysplastic syndrome, *AML* acute myeloid leukaemia, *RAEB* refractory anaemia with excess blasts, *AZA* azacitidine, *PB* peripheral blood, *ECOG PS* Eastern Cooperative Oncology Group performance status, *IPSS* International Prognostic Scoring System, *RBC* red blood cell, *TD* transfusion-dependent, *PLT* platelet, *Hb* haemoglobin, *WBC* white blood cell, *ANC* absolute neutrophil count

When patients were reclassified according to FAB, 131, 101 and 111 patients had MDS-RAEB, MDS-RAEB-t and AML, respectively.

### Azacitidine front-line in AML20–30 vs AML30+

Baseline characteristics were compared in patients with AML30+ and AML20–30 treated with azacitidine front-line. There was no significant difference in median age, gender distribution, presence of MRF, frequency of therapy-related AML, median peripheral blood blasts, Eastern Cooperative Oncology Group performance status (ECOG PS), IPSS cytogenetic risk group, red blood cell (RBC) and platelet (PLT) transfusion dependence (TD), median haemoglobin (Hb) levels and PLT, white blood cell (WBC) or absolute neutrophil count (ANC) (Table 1).

Azacitidine treatment characteristics were also compared between cohorts. No significant difference was observed for the median number of treatment cycles received or median azacitidine dose per cycle (Table 2). The percentage of patients receiving ≥6 cycles, ≥12 cycles, 7 days of azacitidine and the EMA target dose of 75 mg/m^2^ d1-7 did not differ significantly between cohorts and neither did patient status at data cut-off (Table 2). The median number of cycles for patients with AML30+ vs AML20–30 was 5 vs 6 cycles for the total subgroups, 10 vs 9 cycles for responding patients and 2 vs 3.5 cycles for non-responding patients, respectively (Table 2). However, a trend towards a higher percentage of patients receiving fewer than 3 cycles of azacitidine was observed in the AML30+ subgroup (29 vs 17 %, *p* = 0.069; Table 2). This may reflect the observations that adverse events (AEs) were more often the cause for azacitidine discontinuation (12 vs 4 %; *p* = 0.045; Table 2), grade 3–4 infections occurred more often (50 vs 32 %; *p* = 0.047) and there was a trend for higher rates of febrile neutropenia (23 vs 13 %; *p* = 0.075) and 60-day mortality (15 vs 6 %; *p* = 0.053) in the AML30+ subgroup compared with AML20–30 (Table 3).

**Table 2:** Comparison of treatment characteristics of patients with WHO-MDS or WHO-AML receiving azacitidine front-line

	AML30+ (*n* = 111)	AML20–30 (*n* = 79)	*p* value	AML20–30 (*n* = 79)	RAEB-II (*n* = 96)	*p* value	RAEB-II (*n* = 96)	RAEB-I (*n* = 53)	*p* value
Median AZA cycles, n (range) Responders Non-responders	5 (1–51) 10 (2–51) 2 (1–13)	6 (1–49) 9 (1–49) 3.5 (1–28)	0.763 0.818 0.522	6 (1–49) 9 (1–49) 3.5 (1–28)	5 (1–96) 9 (3–81) 3 (1–31)	0.763 1 0.845	5 (1–96) 9 (3–81) 3 (1–31)	7 (1–66) 9 (4–66) 5 (1–12)	0.564 1 0.480
Average AZA cycles^a^, n <3 cycles, % ≥6 cycles, % ≥12 cycles, %	8.5 28.8 49.5 25.2	9.1 16.5 55.7 25.3	0.888 0.069 0.546 1	9.1 16.5 55.7 25.3	9.3 20.8 49.0 22.9	0.964 0.481 0.512 0.729	9.3 20.8 49.0 22.9	8.6 13.2 58.5 26.4	0.869 0.192 0.360 0.618
AZA 7 days^a^, %	75.4	78.9	0.779	78.9	69.2	0.426	69.2	71.1	0.872
EMA target dose^a, b^, %	63.5	62.8	0.950	62.8	59.0	0.730	59.0	43.4	0.123
Median AZA dose/cycle^a^, mg Dose/d, mg	882 130	910 135	0.508 0.759	910 135	905 136	0.906 0.950	905 136	882 130	0.586 0.713
Reason for AZA discont., % Adverse event Death Progressive disease/relapse No response Allo-SCT Patient wish/others Still on AZA at study closure	11.7 32.4 23.4 8.1 1.8 14.4 9.4	3.8 25.3 27.8 10.1 2.5 21.5 6.3	0.045 0.350 0.539 0.639 0.736 0.236 0.434	3.8 25.3 27.8 10.1 2.5 21.5 6.3	3.1 17.7 19.8 12.5 3.1 17.7 24.0	0.790 0.247 0.250 0.614 0.800 0.544 0.001	3.1 17.7 19.8 12.5 3.1 17.7 24.0	1.9 11.3 30.2 7.5 13.2 18.9 17.0	0.592 0.235 0.141 0.264 0.012 0.843 0.274
Patient status, % Dead at data cut-off Alive at data cut-off Unknown	87.5 12.5 0.0	83.5 16.4 0.0	0.700 0.468 1	83.5 16.4 0.0	49.0 51.1 0.0	0.003 <0.001 1	49.0 51.1 0.0	52.8 47.2 0.0	0.706 0.694 1

^a^Regards total azacitidine (AZA) cycles applied to the whole cohort (*n* = 508 [RAEB-I], *n* = 893 [RAEB-II], *n* = 715 [AML20–30], *n* = 111 [AML30+])

^b^75 mg/m^2^ d1–7

*WHO* World Health Organization, *MDS* myelodysplastic syndrome, *AML* acute myeloid leukaemia, *RAEB* refractory anaemia with excess blasts, *EMA* European Medicines Agency, *allo-SCT* allogeneic stem cell transplant, *d* day

**Table 3:** Comparison of TEAEs^a,b^ and response of WHO MDS and WHO AML patients receiving azacitidine front-line

TEAEs^a^	AML30+ (*n* = 111)	AML20–30 (*n* = 79)	*p* value	AML20–30 (*n* = 79)	RAEB-II (*n* = 96)	*p* value	RAEB-II (*n* = 96)	RAEB-I (*n* = 53)	*p* value
TE-thrombocytopenia^b^ G3–4, %	40.5	41.8	0.885	41.8	35.4	0.466	35.4	39.6	0.628
TE-neutropenia^b^ G3–4, %	35.3	39.2	0.488	39.2	32.3	0.414	32.3	41.5	0.284
TE-anaemia^b^ G3–4, %	47.8	53.2	0.591	53.2	54.2	0.924	54.2	47.2	0.487
Febrile neutropenia, %	23.4	12.7	0.075	12.7	8.3	0.337	8.3	10.3	0.643
Infections G3–4, %	49.5	31.6	0.047	31.6	22.9	0.239	22.9	20.8	0.751
Response									
ORR (ITT), % CR CyCR CRi PR	33.4 13.5 5.4 1.8 18.0	35.4 16.5 10.1 3.8 15.2	0.810 0.584 0.233 0.398 0.627	35.4 16.5 10.1 3.8 15.2	25.0 5.2 5.2 6.3 13.5	0.181 0.015 0.210 0.431 0.751	25.0 5.2 5.2 6.3 13.5	30.2 9.4 1.9 5.7 15.1	0.484 0.272 0.216 0.862 0.764
HI without marrow response^c^, %	22.5	16.5	0.337	16.5	24.0	0.239	24.0	32.1	0.279
RBC-TI^c^, %	43.6	42.1	0.872	42.1	28.3	0.100	28.3	58.3	<0.001
PLT-TI^c^, %	38.7	47.1	0.365	47.1	43.3	0.689	43.3	58.3	0.137
Outcome									
30-day mortality, %	8.1	6.3	0.635	6.3	3.1	0.297	3.1	0.0	0.078
60-day mortality, %	15.3	6.3	0.053	6.3	5.2	0.746	5.2	1.9	0.216
1-year survival (total cohort), %	49.6	55.7	0.552	55.7	70.8	0.179	70.8	81.1	0.403
Median overall survival, months	10.9	13.1	0.238	13.1	18.9	0.010	18.9	23.7	0.302

^a^TEAEs were defined as new or worsening AEs between the time of first dose to the end of the safety follow-up period (28 days after the last dose of azacitidine)

^b^TE haematological toxicity was calculated from differential blood counts and transfusions status of all cycles for each patient (no missing data)

^c^Haematological improvement (HI) and achievement of transfusion independence (TI) was assessed according to IWG 2006 criteria. HI and TI are not considered as a form of response in the current AML response criteria but were additionally assessed in AML patients, in order to compare the efficacy of azacitidine across disease entities

*TEAE* treatment emergent (TE) adverse event (AE), *WHO* World Health Organization, *MDS* myelodysplastic syndrome, *AML* acute myeloid leukaemia, *RAEB* refractory anaemia with excess blasts, *G* grade, *ORR* overall response rate, *ITT* intent-to-treat, *CR* complete response, *CyCR* complete cytogenetic response, *CRi* CR with incomplete blood count recovery, *PR* partial response, *RBC* red blood cell, *PLT* platelet, *IWG* International Working Group

**Fig. 1:**
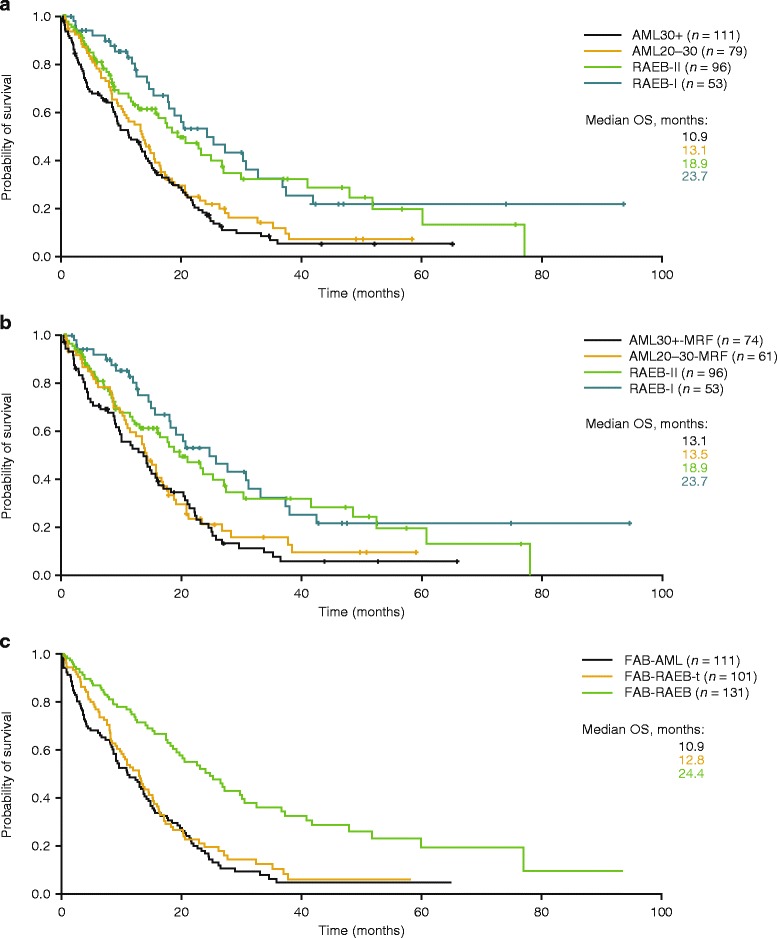
Overall survival of MDS and AML patients treated with azacitidine front-line within the AAR. **a** Patients classified according to WHO criteria. **b** Patients with MRF classified according to WHO criteria. **c** Patients classified according to FAB criteria

Overall response rate (ORR; defined as CR, complete cytogenetic response [CyCR], CR with incomplete blood count recovery [CRi], partial response [PR]), haematological improvement (HI) and achievement of transfusion independence (TI) were similar between patients with AML30+ and AML20–30; 30-day mortality rates were low (8 vs 6 %); 1-year survival rates (50 vs 56 %) and median OS (10.9 vs 13.1 months) were relatively high and did not differ significantly between AML30+ and AML20–30, respectively (Tables 3 and 4 and Fig. 1a). This remained true when analysing patients with MRF (13.1 vs 13.5 months, *p* = 0.474; Table 5 and Fig. 1b).

**Table 4:** WHO classification: OS of patients with MDS or AML receiving azacitidine front-line

WHO diagnosis	*n*	Median OS, mo	95 % CI, mo	*p* value
AML30+ AML20–30 MDS-RAEB-II MDS-RAEB-I	111 79 96 53	10.9 13.1 18.9 23.7	7.5–14.3 9.8–16.5 12.7–25.1 14.4–33.0	<0.001^a^
AML30+ AML20–30	111 79	10.9 13.1	7.5–14.3 9.8–16.5	0.238
AML20–30 MDS-RAEB-II	79 96	13.1 18.9	9.8–16.5 12.7–25.1	0.010^b^
MDS-RAEB-II MDS-RAEB-I	96 53	18.9 23.7	12.7–25.1 14.4–33.0	0.302

^a^HR = 1.292; 95 % CI 1.168, 1.430

^b^HR = 1.645; 95 % CI 1.123, 2.409

**Table 5:** WHO Classification: OS of patients with MDS or AML-MRF receiving AZA front-line

WHO diagnosis	*n*	Median OS, mo	95 % CI, mo	*p* value
AML30-MRF AML20–30-MRF MDS-RAEB-II MDS-RAEB-I	74 61 96 53	13.1 13.5 18.9 23.7	8.6–17.5 10.5–16.5 12.7–25.1 14.4–33.0	0.001^a^
AML30-MRF AML20–30-MRF	74 61	13.1 13.5	8.6–17.5 10.5–16.5	0.474
AML20–30-MRF MDS-RAEB-II	61 96	13.5 18.9	10.5–16.5 12.7–25.1	0.033^b^
MDS-RAEB-II MDS-RAEB-I	96 53	18.9 23.7	12.7–25.1 14.4–33.0	0.302

^a^HR = 1.247; 95 % CI 1.118–1.392

^b^HR = 1.551; 95 % CI 1.032–2.331

*WHO* World Health Organization, *OS* overall survival, *MDS* myelodysplastic syndrome, *AML* acute myeloid leukaemia, *MRF* MDS-related features, *AZA* azacitidine, *CI* confidence interval, *RAEB* refractory anaemia with excess blasts, *HR* hazard ratio

### Impact of MDS-related features in AML

**Fig. 2:**
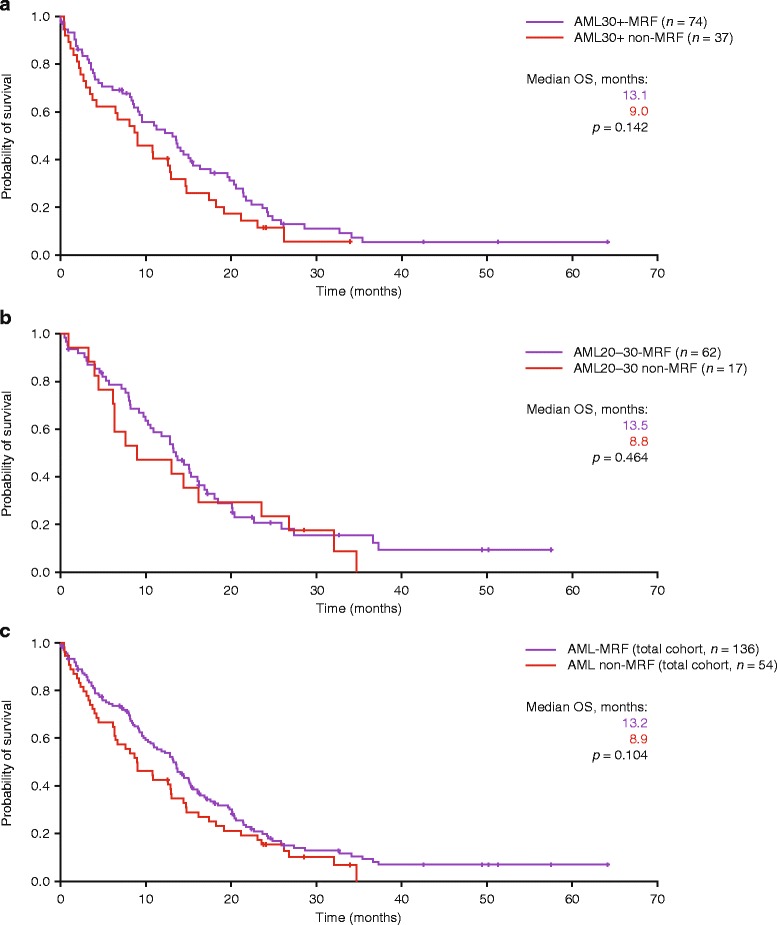
Effect of MRF on OS of patients with **a** AML30+, **b** AML20–30 and **c** AML (all patients)

No negative impact was observed for the presence of MRF in AML patients, irrespective of bone marrow blast count. Median OS was 13.1 vs 9.0 months (*p* = 0.142) for patients with AML30+ with vs without MRF; 13.5 vs 8.8 months (*p* = 0.464) for patients with AML20–30 with vs without MRF; and 13.2 vs 8.9 months (*p* = 0.104) for all AML patients with vs without MRF (Fig. 2a–c).

### Impact of the WHO classification on the distinction between MDS and AML

In order to see whether we could validate the WHO classification in patients treated with azacitidine front-line, we compared baseline and treatment characteristics, as well as the occurrence of treatment-emergent AEs (TEAEs), response, and outcomes in patients with AML20–30 and MDS-RAEB-II, as well as between patients with MDS-RAEB-II and MDS-RAEB-I, in addition to the comparison between AML30+ and AML20–30 detailed above (Tables 1, 2, 3, 4 and 5).

The AML20–30 cohort had a higher proportion of patients older than 75 years (60 vs 39 %; *p* = 0.034), patients had a higher PLT count (66 vs 44G/L; *p* = 0.036) and a trend for a higher proportion of patients with elevated baseline peripheral blood blasts (65 vs 45 %; *p* = 0.058) than the RAEB-II cohort, respectively. No significant differences in baseline characteristics could be found when comparing the MDS-RAEB-II with the MDS-RAEB-I cohort (Table 1). There were no differences in treatment characteristics or the occurrence of TEAEs between patients with AML20–30 and MDS-RAEB-II or between MDS-RAEB-II and MDS-RAEB-I, respectively (Tables 2 and 3). However, in comparison with patients with AML20–30, a significantly higher proportion of patients with MDS-RAEB-II were still on azacitidine (6 vs 24 %, *p* = 0.001), still alive (16 vs 51 %, *p* < 0.001) and correspondingly fewer had died (84 vs 49 %; *p* = 0.003) at study cut-off, respectively (Table 2). No such differences were observed between the MDS-RAEB-II and MDS-RAEB-I cohorts, respectively (Table 2).

Although ORR and HI were comparable between all four cohorts, a higher complete response (CR) rate was observed in AML20–30 compared with MDS-RAEB-II (17 vs 5 %, *p* = 0.015), which may reflect the presence of residual myelodysplasia in patients with MDS: bone marrow blast count reduction to <5 %, normalisation of peripheral blood cell counts and lack of dysplasia are required for the definition of CR in MDS according to International Working Group (IWG) 2000 [27], but not for the definition of CR in AML [28]. However, the implications of residual dysplasia in patients with MDS or AML who otherwise meet the criteria for CR remain unclear [13, 28].

Despite higher CR rates in AML20–30, median OS was significantly worse as compared with MDS-RAEB-II (13.1 vs 18.9 months, *p* = 0.010; Tables 3 and 4; Fig. 1a). The same held true when considering only those AML patients that had MRF (13.5 vs 18.9 months; *p* = 0.033; Table 5 and Fig. 1b). However, OS of those patients that did achieve CR was much longer for patients with RAEB-II (59.0 months), as compared to patients with AML20–30 achieving CR (18.4 months).

Why higher CR rate in AML20–30 did not translate into longer OS of the total cohort remains speculative at this time point. It was stated by IWG in 2006 that ‘although it is logical that a complete cytogenetic response, as in AML, would also prolong survival in MDS, there are presently little data to support this assumption’ [13]. Furthermore, it is becoming widely accepted that while CR remains the main treatment goal in elderly MDS and AML patients treated with hypomethylating agents, it does not seem to be a prerequisite for survival benefit in MDS (e.g. Gore SD, Haematologica 2013) or AML [24, 29, 30]. Previous reports of MDS patients treated with intensive chemotherapy [31–33] or decitabine [34] have also observed higher CR rates for patients with RAEB-t compared with other ‘lower-risk’ MDS patients. The shorter OS (9.0 vs 16.6 months; *p* = 0.021), despite higher CR rates in RAEB-t as compared with other types of MDS observed in the latter analysis, may be due to shorter duration of response (5.0 vs 9.9 months; *p* = 0.024) [34]. The same group also observed shorter OS (13.9 vs 18.6 months, *p* = 0.022) despite similar response rates (27 vs 27 %, *p* > 0.05) in MDS patients that transformed to AML, compared to those that did not [35]. One must also bear in mind that CR only occurs in a minority of patients treated with hypomethylating agents. Therefore, small differences in CR rates (16.5 % in AML20-30 vs 5.2 % in MDS-RAEB-II), even if they are statistically significant, do not necessarily have to translate into longer median OS for the whole cohort. The adverse prognosis of having AML (rather than MDS) thus seems to outweigh the benefits of slightly higher CR rate.

In patients with MDS-RAEB-I, we observed a higher proportion of patients with the intention to proceed to allogeneic stem cell transplantation (*p* = 0.012; Table 2), a higher proportion of patients achieving RBC-TI (*p* < 0.001; Table 3) and a trend for lower 30-day mortality (*p* = 0.078; Table 3) compared with patients with MDS-RAEB-II; 1-year survival rates and median OS were not significantly different between these two subgroups (Tables 3 and 5; Fig. 1a). Time to treatment from first cytopenias or first diagnosis was shorter for patients with AML compared with MDS (Additional file 3: Table S3). Median cycles to the first response or best response, as well as duration of response, were similar for all four disease categories. However, patients with MDS-RAEB-I seemed to have longer relapse- and event-free survival, as well as time from azacitidine stop to death, than patients with MDS-RAEB-II, AML20–30 or AML30+, respectively (Additional file 3: Table S3).

### Impact of the FAB classification on the distinction between MDS and AML

When patients were classified according to FAB criteria, patients with RAEB-t had similar survival to patients with AML30+ (12.8 vs 10.9 months, *p* = 0.376), but significantly worse OS than patients with MDS-RAEB (12.8 vs 24.4 months, *p* < 0.001; Table 6; Fig. 1c).

**Table 6:** FAB classification: OS of patients with MDS or AML receiving azacitidine front-line

FAB diagnosis	*n*	Median OS, mo	95 % CI, mo	*p* value
AML30+ MDS-RAEB-t MDS-RAEB	111 101 131	10.9 12.8 24.4	7.5–14.3 10.1–15.5 18.5–30.3	<0.001^a^
AML30+ MDS-RAEB-t	111 101	10.9 12.8	7.5–14.3 10.1–15.5	0.376
MDS-RAEB-t MDS-RAEB	101 131	12.8 24.4	10.1–15.5 18.5–30.3	<0.001^b^

^a^HR = 1.248; 95 % CI 1.249, 1.634

^b^HR = 2.185; 95 % CI 1.557, 3.066

*FAB* French-American-British, *OS* overall survival, *MDS* myelodysplastic syndrome, *AML* acute myeloid leukaemia, *CI* confidence interval, *RAEB* refractory anaemia with excess blasts, *HR* hazard ratio

## Discussion

To date, no clinical trial has been performed that specifically assessed the efficacy of azacitidine in the AML patient subgroup for which the drug was initially approved, namely AML20–30 (formerly MDS-RAEB-t). Depending on the classification system used to distinguish MDS from AML, AML20–30 was either grouped together with high-risk MDS (FAB classification) or AML (WHO classification). The only available clinical trial data specific to the AML20–30 subgroup of patients treated with azacitidine is based on sub-analyses of the AZA-MDS-001 trial and the CALGB protocol 8421, which had low AML20–30 patient numbers (*n* = 53 and *n* = 24) [23, 36]. In addition, one retrospective analysis from the Dutch named patient programme published data on 38 AML20–30 patients treated with azacitidine, but 13 % of these had relapsed/refractory AML [37]. We therefore present data from the largest cohort of patients with AML20–30 treated with azacitidine front-line (*n* = 79) to date. Outcomes were encouraging with an ORR of 34 % and a median OS of 13.1 months, especially taking the advanced age of this cohort into consideration (median age 77 years with 60 % >75 years). In comparison, patients with AML20–30 included within former clinical trials were younger (median ages were 65, 70 and 72 years in the CALGB-8421 protocol, AZA-MDS-001 trial and the Dutch named patient programme, respectively) [23, 36, 37]. As a side note, the median OS of 24.5 months obtained in patients with AML20–30 in the AZA-MDS-001 trial is exceptionally long [36], and thus far, no other group has been able to find similarly long OS times in elderly AML patients, no matter which treatment was investigated, and no matter which bone marrow blast count was used as cut-off (Table 7 [24, 38–48]). Median OS of patients receiving conventional care regimen in the AZA-MDS-001 trial was also extraordinarily high (16.0, 17.0, 14.2 and 13.4 months for all conventional care regimen combined, low-dose cytarabine, IC and best supportive care (BSC), respectively) [36], indicating that patient selection may have favoured improved survival. The lack of clinical trials allowing direct comparison of the efficacy of azacitidine in AML20–30 vs AML30+ is likely due to the requirements imposed by the registration agencies. A randomised trial performed exclusively in AML30+ [24] was requested in order to widen the registration indication of azacitidine to include AML30+, thus eliminating the possibility of a direct comparison of the efficacy of azacitidine in AML20–30 vs AML30+. Our data show the first direct comparison of these two patient groups and reveal similar baseline and treatment characteristics, ORR and OS for patients with AML20–30 (*n* = 79) vs AML30+ (*n* = 111) treated with azacitidine front-line, respectively. We further confirm the efficacy of azacitidine in the subset of patients with AML30+, with a median OS of 10.9 months observed in our cohort (*n* = 111), which was similar to that observed in the recently published phase III clinical trial AML-001 (10.4 months; *n* = 241) [24]. Thus, patients with AML30+ seem to derive similar clinical benefit from azacitidine in terms of OS prolongation, as patients with AML20–30.

**Table 7:** Elderly AML front-line treatment options and median OS times

Treatment	*N*	Age, years	Median age, years	CR/CRi, %	OS, mo	Phase	Ref
Untreated	3367	≥65	77	n.g.	2	Retrosp.	[39]
HU+/-ATRA	99^a^	>60	74	1	~3	III	[43]
HD-LEN	33	>60	71	30	4	II	[45]
LD-AraC+/-ATRA	103^a^	>60	74	18	<5	III	[43]
CFA	112	>60	71	46	9.4	II	[40]
CFA + LD-AraC	54	>60	71	63	11.4	II	[42]
CFA + LD-AraC/DAC	60	>60	70	58	12.7	II	[44]
CFA + LD-AraC/DAC	118	>60	68	60	11.1	II	[44]
Allo-SCT	46	≥65	67	n.g.	22	Retrosp.	[39]
Intensive CTX	1856	≥65	74	n.g.	6	Retrosp.	[39]
Intensive CTX (3 + 7)	416	>65	67	57	12	III	[41]
BSC ↔ allo-SCT	352	≥60	n.g.	n.g.	9.0	Retrosp.	[47]
BSC ↔ allo-SCT	5480	≥65	78	n.g.	3.0	Retrosp.	[39]
DAC (DACO-16)	238	>65	74	18	7.7	III	[48]
AZA (AZA-AML-001)	241	>65	75	28	10.4	III	[24]
AZA-AAR	193	>17	77	18	12.6	Retrosp.	[46]
AZA + LEN	42	>60	74	28	15.9	I/II	[38]

^a^Included 14 patients with high-risk MDS

*n.g.* *CR* complete response, *CRi* CR with incomplete blood count recovery, *OS* overall survival, *n.g.* not given, *HU* hydroxyurea, *ATRA* all trans retinoic acid, *HD* high-dose, *LEN* lenalidomide, *LD-AraC* low-dose cytarabine, *CFA* clofarabine, *DAC* decitabine, *allo-SCT* allogeneic stem cell transplantation, *CTX* chemotherapy, *BSC* best supportive care, *AZA* azacitidine, *AML* acute myeloid leukaemia, *AAR* Austrian Azacitidine Registry, *MDS* myelodysplastic syndrome

In AML patients, the presence of MRF has been shown to be associated with adverse clinical outcome [49–51]. Although previous studies reporting on azacitidine in the front-line setting included patients with MRF, outcomes were not reported separately. We demonstrate for the first time that the presence of MRF has no adverse effect on OS of elderly patients treated with azacitidine front-line (Fig. 2a–c). This seems to be of clinical relevance in light of adverse outcomes observed with front-line IC in patients with secondary AML compared with de novo AML (6.8 vs 14.8 months; *p* < 0.05 [52]; 8.6 vs 23.0 months; *p* < 0.001 [53]). Another trial performed in patients with AML-MRF reported a median OS of only 14 months in young patients (<60 years) treated with an IC regimen [54]. The median OS of 13.2 months observed in our elderly AML-MRF patients (median age 77 years) indicates that these patients benefit from treatment with azacitidine (Fig. 2c).

We also present the first direct comparison of baseline factors, treatment-related factors and outcomes of patients with MDS-RAEB-I, MDS-RAEB-II, AML20–30 and AML30+ treated with azacitidine front-line (Tables 1, 2, 3, 4 and 5). Our data indicate that patients with AML20–30 have comparable baseline, treatment and response characteristics to patients with MDS-RAEB-II or AML30+ but behave more like AML30+ than MDS-RAEB-II with respect to OS (Table 4; Fig. 1a). This implies that the WHO reclassification of patients with 20–30 % bone marrow blasts from MDS-RAEB-t to AML seems appropriate in patients receiving azacitidine as front-line agent. Near identical observations have been made in patients treated with decitabine in a pooled sub-analysis of two clinical trials, which demonstrated significantly shorter median OS in patients with AML20–30 compared with patients with higher-risk MDS (9.0 vs 16.6 months; *p* = 0.021), respectively [34].

The effect of time from diagnosis to treatment (TDT) on overall survival of patients with MDS and AML remains obscure. In high-risk MDS patients including RAEB-t (treated with chemotherapy, azacitidine, dectiabine, lenalidomide or others), median TDT varied from 4.8 months [55] to >1 year [15], whereas separate analyses of RAEB-t and RA/RARS by others revealed a significantly shorter median TDT for RAEB-t (7.3 vs. 18.3 months, *p* = 0.021) [34], and others found a significantly shorter TDT for those MDS patients that eventually transformed to AML (10.8 months) as compared with those who did not (20.8 months) [35]. These reports do not allow conclusions as to why shorter TDT have been observed in patients with higher-risk MDS and RAEB-t. One might speculate, however, that these patients are perceived as being in more dire need of treatment (due to higher bone marrow blasts, worse cytopenias and/or adverse cytogenetics) and are more likely to receive treatment soon after initial diagnosis. This is backed up by our own observations, which show a progressively shorter time from initial cytopenias (4.2, 2.8, 1.6 and 0.9 months) as well as initial diagnosis (3.0, 1.6, 0.6 and 0.5 months) to azacitidine treatment start for patients with RAEB-I, RAEB-II, AML20–30 and AML30+, respectively (Additional file 3: Table S3). Whether earlier treatment initiation in higher-risk MDS and AML20–30 translates into earlier response, longer response duration, or possibly results in a survival advantage, remains unknown at this time point. Even in the event that a correlation between TDT and outcome could be shown, it would still need to be clarified, whether this might also reflect a more aggressive underlying biology and kinetics of the disease.

There are not many studies that address the topic of TDT in AML, but those that did were all performed exclusively in patients treated with intensive chemotherapy approaches, with (partially) controversial results [53, 56–59]. Most results however, do indicate that longer TDT is associated with worse prognosis, i.e. lower response rates and shorter OS [56–59], and it was concluded that initiating therapy as soon as possible after diagnosis might be a potential strategy to improve OS in AML patients [59]. Whether this can be translated to patients treated with hypomethylating agents remains to be shown.

In this report, we have shown that patients with AML20–30 treated with azacitidine front-line should be regarded as ‘true AML’. In line with the above [56–59], we believe that treatment should thus be initiated without delay.

For decades, it has remained controversial whether AML20–30 (formerly MDS-RAEB-t) potentially follows a more benign disease trajectory than AML30+ and whether consideration should be given to possible retention of MDS-RAEB-t as a separate disease entity distinct from MDS and AML [7, 16–19, 22, 31, 33, 34, 60–69]. The fact that the term ‘RAEB-t’ is still used in very recent reports on the efficacy of decitabine in patients with RAEB-t [34, 70] shows the actuality of this issue. In an effort to clarify this matter for elderly patients treated with azacitidine front-line, we reclassified our cohort according to the FAB classification. Patients with MDS-RAEB-t had significantly worse OS than patients with MDS-RAEB, but similar survival to patients with AML30+ (Table 6; Fig. 1c). We thus show that the FAB disease category MDS-RAEB-t does not adequately distinguish risk categories or behave as an entity distinct from both MDS and AML with regard to patient outcome. In contrast, and as mentioned above, the WHO classification of MDS and AML adequately distinguished patient categories with distinct outcomes (13.1 vs 18.9 months for AML20–30 vs MDS-RAEB-II, *p* = 0.010; Table 4 and Fig. 1a). We conclude that the elimination of the FAB category RAEB-t, and its incorporation within the WHO categories MDS-RAEB-II and AML, seems justified in elderly AML patients treated with azacitidine front-line.

In our cohort, the distinction between MDS-RAEB-I or MDS-RAEB-II could not separate groups with differing OS (23.7 vs 18.9 months, *p* = 0.302; Table 4 and Fig. 1a). Of note, the IPSS [8] gave more weight to the bone marrow blast threshold between 10 and 11 % (+1.0 additional score points for 11–20 %) than to the threshold between 20 and 21 % (+0.5 additional score points). Similarly, the revised IPSS [71] conceded 1 additional score point for patients with ≥11 % bone marrow blasts but gave no further weight to bone marrow blast percentages >11 % (0 additional score points). Thus, while we could not confirm the capability of the WHO classification of MDS-RAEB-I and MDS-RAEB-II for distinguishing differing risk categories, we could confirm the weighting for bone marrow blasts chosen by the IWG for the prognosis of MDS.

## Conclusions

We have demonstrated (i) the promising potential of azacitidine as front-line treatment for patients with AML, irrespective of bone marrow blast count and/or presence of MRF; (ii) the adequate categorisation of patients with 20–30 % bone marrow blasts as AML and (iii) we have addressed some topics of the WHO 2008 classification of MDS and AML; in particular, we confirm the validity of the WHO classification of MDS and AML, and that the former FAB category MDS-RAEB-t was correctly consolidated under the diagnosis of AML in patients treated with azacitidine front-line. Patients with AML20–30 should thus be regarded as ‘true AML’, and in our opinion, treatment should be initiated without delay. Our results should thus pave the way for the accurate classification and prognostication, as well as earlier treatment initiation of elderly patients with AML20–30.

## Methods

### Patient population

The AAR of the ‘Arbeitsgemeinschaft Medikamentöse Tumortherapie’ (AGMT) Study Group is a multicentre database, initiated to gain a comprehensive view of the use, safety and efficacy of azacitidine in a broad range of patients with MDS or AML in a ‘real-world’ clinical practice setting (ClinicalTrials.gov: NCT01595295) [61, 62, 72–80]. Ethics Committee approval was obtained on February 06, 2009. Seventeen centres participated in the registry. The only inclusion criteria were a diagnosis of MDS or AML according to WHO criteria and treatment with at least one dose of azacitidine. No formal exclusion criteria existed, as the aim was to include all patients treated with azacitidine, irrespective of age, comorbidities and/or number of previous lines of treatment. Treatment indication and the decision to offer azacitidine, as well as dose, schedule and dose reductions/escalations were exclusively based on the risk/benefit estimation of the treating physician. Due to a lack of treatment alternatives, AML patients with >30 % bone marrow blasts were also offered treatment with azacitidine. They were informed of off-label use and gave written informed consent. Data were entered into electronic case report forms (eCRFs) by physicians and/or trained clinical trial personnel at the respective centres. All eCRFs were monitored centrally in order to ensure data integrity and plausibility. Missing data were low. If necessary, centres received queries specifying incomplete data or questions to reconfirm data.

This analysis selected patients with MDS-RAEB-I/II or AML receiving azacitidine as front-line therapy, which was defined as absence of prior disease-modifying treatment. Only prior treatment with growth factors (granulocyte-colony stimulating factor, erythropoietin, thrombopoietin-stimulating agents) and/or prior iron chelators was allowed. Patients treated with prior hydroxyurea, immunosuppressive treatment (cyclosporin A, ATG), low-dose cytarabine, revlimid, thalidomide, tyrosine kinase inhibitors and/or intensive chemotherapy for MDS or AML were considered as pretreated. Assessment of response, safety and endpoints, and statistical analyses within the AAR were performed as previously described [61, 62, 73]. The IPSS cytogenetic risk score was established for and validated in patients with MDS and is not commonly used to stratify cytogenetic risk in AML patients. However, we used this score for both MDS and AML patients, in order to be able to compare frequencies of certain karyotypes (not actual cytogenetic risk) across these patient groups. Response was assessed according to current criteria. ORR included CR, CyCR, CRi and PR. HI and achievement of TI were assessed according to IWG 2006 criteria [13]. HI and TI are considered to be a form of response in MDS [13], but not in the current AML response criteria [28, 81]. HI and TI were, however, also assessed in AML patients, in order to compare the (putative) efficacy of azacitidine across disease entities, as has been done by several clinical trials [23, 36]. Adverse events were assessed according to the Common Terminology Criteria for adverse events (CTCAEv.4) (http://evs.nci.nih.gov/ftp1/CTCAE/About.html). TEAEs were defined as new or worsening AEs between the time of first dose of azacitidine to the end of the safety follow-up period (28 days after the last dose of azacitidine). Treatment-emergent haematological toxicity was calculated from differential blood counts and transfusions status of all cycles for each patient (no missing data).

### Statistics

OS as of azacitidine start was assessed using the Kaplan–Meier method. Data-cleaning and survival-analysis cut-off date was 18 June 2015, for AML patients and 17 August 2015, for MDS patients. Univariate analyses were performed with log-rank tests. Cox-regression stratified on the various factors was used for univariate analyses of risk-factors for OS. Baseline characteristics were compared using the chi-squared test. Analyses were performed with SPSS. No adjustments were made for multiple testing.

### Ethics, consent and permissions

Ethics Committee approval by the ‘Ethikkommission für das Bundesland Salzburg’ was obtained on 06 February 2009 (reference number 415-EP/39/11-2009).

### Consent to publish

Written informed consent to allow the collection of personal data, and thus, participation in the analysis and ultimately data publication was obtained for retrospectively documented patients who were alive, as well as for prospectively included patients.

## Additional files

Additional file 1: Table S1. FAB and WHO classifications of MDS and AML. (DOC 37 kb) Additional file 2: Table S2. Treatment after azacitidine in young patients. (DOC 40 kb) Additional file 3: Table S3. Comparison of time to treatment, time to response, response duration, RFS, EFS, and time to death from AZA stop of patients with MDS or AML according to WHO classification receiving AZA front-line within the AAR. (DOC 37 kb)

## Abbreviations

AAR: Austrian Azacitidine Registry
AE: adverse event
AGMT: Arbeitsgemeinschaft Medikamentöse Tumortherapie
allo-SCT: allogeneic stem cell transplantation
AML: acute myeloid leukaemia
ANC: absolute neutrophil count
ATRA: all trans retinoic acid
AZA: azacitidine
BSC: best supportive care
CFA: clofarabine
CI: confidence interval
CR: complete response
CRi: CR with incomplete blood count recovery
CTX: chemotherapy
CyCR: complete cytogenetic response
DAC: decitabine
ECOG PS: Eastern Cooperative Oncology Group performance status
eCRF: electronic case report form
EFS: event-free survival
EMA: European Medicines Agency
FAB: French-American-British
FDA: Food and Drug Administration
Hb: haemoglobin
HD: high-dose
HI: haematological improvement
HU: hydroxyurea
IC: intensive chemotherapy
IPSS: International Prognostic Scoring System
ITT: intent-to-treat
IWG: International Working Group
LD-AraC: low-dose cytarabine
LEN: lenalidomide
MDS: myelodysplastic syndrome
MRC: MDS-related changes
MRF: MDS-related features
NCCN: National Comprehensive Cancer Network
n.g.: not given
ORR: overall response rate
OS: overall survival
PB: peripheral blood
PLT: platelet
PR: partial response
RAEB: refractory anaemia with excess blasts
RAEB-t: RAEB in transformation
RBC: red blood cell
RFS: relapse-free survival
TD: transfusion-dependent
TEAE: treatment-emergent AE
TI: transfusion-independent
WBC: white blood cell
WHO: World Health Organization
