# Supplementary material for: Azacitidine front-line in 339 patients with myelodysplastic syndromes and acute myeloid leukaemia: comparison of French-American-British and World Health Organization classifications
Source: J Hematol Oncol. 2016 Apr 16;9:39. doi: 10.1186/s13045-016-0263-4 (PMC4833933; doi:10.1186/s13045-016-0263-4)
Supplement: Additional file 1: Table S1. — FAB and WHO classifications of MDS and AML. (DOC 37 kb) [file 13045_2016_263_MOESM1_ESM.doc]

Additional file 1: Table S1. FAB and WHO-classifications of MDS and AML.

| FAB-classification | PB anomalies | BM anomalies |
| --- | --- | --- |
| RAEB | - Cytopenia(s) - 2–<5% blasts - No Auer rods - Monocytes <1.000/µL | - Unilineage or multlineage dysplasia - 5–20% blasts - No Auer rods - Ringed sideroblasts >15% +/- |
| RAEB-t | - Cytopenia(s) - ≥5% and <30% blasts^1^ - Auer rods +/- - Monocytes >1.000/µL +/- | - Unilineage or multlineage dysplasia - 21–30% blasts - Auer rods +/- - Ringed sideroblasts >15% +/- |
| AML | - Cytopenia(s) - ≥30% blasts^1^ - Auer rods +/- - Monocytes >1.000/µL +/- | - >30% blasts - Auer rods +/- - Ringed sideroblasts >15% +/- |
| WHO-classification | PB anomalies | BM anomalies |
| RAEB-I | - Cytopenia(s) - 2–<5% blasts^2^ - No Auer rods - Monocytes <1.000/µL | - Cytopenia(s) - 2<5% blasts^2^ - No Auer rods - Ringed sideroblasts >15% +/- |
| RAEB-II | - Cytopenia(s) - 5–19% blasts - Auer rods +/-^3^ - Monocytes <1.000/µL | - Unilineage or multlineage dysplasia - 10–19% blasts - Auer rods +/- - Ringed sideroblasts >15% +/- |
| AML | - Cytopenia(s) - ≥20% blasts^1^ - Auer rods +/- - Monocytes >1.000/µL +/- | - ≥20% blasts^4,5^ - Auer rods +/- - Ringed sideroblasts >15% +/- |

^1^In the absence of BM blasts (‘peripheral leukemia’).

^2^If BM blasts <5% but PB blasts are 2–4%, the diagnostic classification is RAEB-I.

^3^Cases with Auer rods and <5% PB blasts and <10% BM blasts should be classified as RAEB-II.

^4^Exceptions to this include leukemias with certain genetic abnormalities, such as those with t(8;21), inv(16), or t(15;17), and myeloid sarcoma, which are considered diagnostic of AML without regard to the BM blast count.

^5^The leukemic cells must be of myeloid origin as demonstrated by either the presence of Auer rods, cytochemical positivity for myeloperoxidase, or presence of sufficient myeloid markers recognized by immunophenotyping.
