# Supplementary material for: Azacitidine front-line in 339 patients with myelodysplastic syndromes and acute myeloid leukaemia: comparison of French-American-British and World Health Organization classifications
Source: J Hematol Oncol. 2016 Apr 16;9:39. doi: 10.1186/s13045-016-0263-4 (PMC4833933; doi:10.1186/s13045-016-0263-4)
Supplement: Additional file 2: Table S2. — Treatment after azacitidine in young patients. (DOC 40 kb) [file 13045_2016_263_MOESM2_ESM.doc]

Additional file 2: Table S2. Treatment after azacitidine in young patients

| RAEB-I | <65 years (n=15) | <60 years  (n=8) | <50 years (n=1) | <40 years  (n=0) |
| --- | --- | --- | --- | --- |
| AZA started for bridging and/or Allo-SCT after AZA, n (%)  High-dose CTX after AZA, n (%)  LDAC/HU after AZA, n (%)  Others, n (%)  Still on AZA, n (%) | 8 (53)  2 (13)  2 (13)  1 (7)  2 (13) | 5 (63)  0 (0)  1 (13)  0 (0)  2 (25) | 1 (100)  0 (0)  0 (0)  0 (0)  0 (0) | 0 (0)  0 (0)  0 (0)  0 (0)  0 (0) |
| RAEB-II | <65 years (n=20) | <60 years  (n=12) | <50 years (n=6) | <40 years  (n=1) |
| AZA started for bridging and/or Allo-SCT after AZA, n (%)  High-dose CTX after AZA, n (%)  Revlimid after AZA, n (%) Others, n (%)  Still on AZA, n (%)  AZA stop due to death, n (%)  AZA stop due to disease progression  No trt. after AZA, n (%)  Lost to follow-up, n (%) | 8 (40)  2 (10)  1 (5)  1 (5)  1 (5)  3 (15)  2 (10)  2 (10) | 6 (50)  1 (8)  1 (8)  0 (0)  0 (0)  1 (8)  1 (8)  1 (8) | 3 (50)  1 (17)  1 (17)  0 (0)  0 (0)  0 (0)  1 (17)  0 (0) | 1 (100)  0 (0)  0 (0)  0 (0)  0 (0)  0 (0)  0 (0)  0 (0) |
| AML20-30 | <65 years (n=6) | <60 years (n=4) | <50 years (n=2) | <40 years  (n=0) |
| AZA started for bridging and/or Allo-SCT after AZA, n (%)  High-dose CTX after AZA, n (%)  HU after AZA, n (%)  Others, n (%)  Still on AZA, n (%) | 1 (17)  2 (33)  1 (17)  1 (17)  1 (17) | 1 (25)  1 (25)  1 (25)  0 (0)  1 (25) | 1 (50)  1 (50)  0 (0)  0 (0)  0 (0) | 0 (0)  0 (0)  0 (0)  0 (0)  0 (0) |
| AML30+ | <65 years (n=9) | <60 years  (n=5) | <50 years (n=3) | <40 years  (n=3) |
| AZA started for bridging and/or Allo-SCT after AZA, n (%)  High-dose CTX after AZA, n (%)  Others, n (%)  AZA stop due to death, n (%)  Still on AZA, n (%) | 4 (44)  2 (22)  1 (11)  2 (22)  0 (0) | 4 (80)  0 (0)  0 (0)  1 (20)  0 (0) | 2 (67)  0 (0)  0 (0)  1 (33)  0 (0) | 2 (67)  0 (0)  0 (0)  1 (33)  0 (0) |
