# Supplementary material for: Azacitidine front-line in 339 patients with myelodysplastic syndromes and acute myeloid leukaemia: comparison of French-American-British and World Health Organization classifications
Source: J Hematol Oncol. 2016 Apr 16;9:39. doi: 10.1186/s13045-016-0263-4 (PMC4833933; doi:10.1186/s13045-016-0263-4)
Supplement: Additional file 3: Table S3. — Comparison of time to treatment, time to response, response duration, RFS, EFS, and time to death from AZA stop of patients with MDS or AML according to WHO classification receiving AZA front-line within the AAR. (DOC 37 kb) [file 13045_2016_263_MOESM3_ESM.doc]

Additional file 3: Table S3. Comparison of time to treatment, time to response, response duration, RFS, EFS, and time to death from AZA stop of patients with MDS or AML according to WHO classification receiving AZA front-line within the AAR

|  | RAEB-I  (n=53) | RAEB-II  (n=96) | AML20–30  (n=79) | AML30+  (n=111) |
| --- | --- | --- | --- | --- |
| OS (months), median^1^  (range) | 23.7  (1.4–92.0) | 18.9  (0.2–75.7) | 13.1  (0.5–57.3) | 10.9  (0.0–63.9) |
| First cytopenias to AZA start (months), median^2^ (range) | 4.2  (0–183.4) | 2.8  (0.0–98) | 1.6  (0.0–82.3) | 0.9  (0.0–85.0) |
| Initial diagnosis to AZA start (months), median^2^ (range) | 3.0  (0–99.8) | 1.6  (0.0–99) | 0.6  (0.0–90.8) | 0.5  (0.0–83.0) |
| Cycles to first response (n), median  (range) | 4  (2–11) | 4  (3–9) | 4  (1–17) | 3  (1–16) |
| Cycles to best response (n), median  (range) | 5  (2–14) | 5  (3–17) | 5  (1–20) | 5  (1–19) |
| Duration of response^3^ (months), median^2^  (range) | 8.5  (0.2–86.0) | 6.2  (0.2–43.6) | 6.0  (0.4–41.9) | 7.6  (0.3–41.7) |
| RFS^4^ all responders^3^ (months), median^2^  (range) | 12.4  (2.2–92.0) | 9.7  (1.4–47.1) | 9.1  (1.9–49.2) | 8.8  (0.9–42.4) |
| EFS^5^ all patients (months), median^2^  (range) | 10.6  (0.0–46.1) | 5.5  (0.0–55.6) | 8.1  (0.0–49.2) | 5.5  (0.0–42.4) |
| AZA stop to death (months), median^2^  (range) | 6.5  (0.0–35.5) | 2.3  (0.3–23.4) | 2.1  (0.0–15.8) | 1.6  (0.0–27.3) |

^1^Estimated according to Kaplan–Meier method

^2^Calculated as true median (not estimated to Kaplan–Meier)

^3^Includes CR, CRi, PR and HI

^4^RFS; Events included treatment failure, progressive disease, relapse after response, new cytogenetic aberration or clonal evolution, or death due to any reason

^5^EFS; Events included treatment failure, progressive disease, relapse after response, new cytogenetic aberration or clonal evolution, or death due to any reason
